# Breaking conversational rules matters to captive gorillas: A playback experiment

**DOI:** 10.1038/s41598-020-63923-7

**Published:** 2020-04-24

**Authors:** Loïc Pougnault, Florence Levréro, Baptiste Mulot, Alban Lemasson

**Affiliations:** 1 Univ Rennes, Normandie Univ, CNRS, EthoS (Éthologie animale et humaine) - UMR 6552, F-35000 Rennes, France; 2 Université de Lyon/Saint-Etienne, CNRS, Equipe Neuro-Ethologie Sensorielle, ENES/CRNL, UMR5292, INSERM UMR_S 1028, Saint-Etienne, France; 3 ZooParc de Beauval & Beauval Nature, 41110 Saint Aignan, France

**Keywords:** Evolution, Zoology

## Abstract

Across human cultures, conversations are regulated by temporal and social rules. The universality of conversational rules suggests possible biological bases and encourages comparisons with the communicative interactions of nonhuman animals. Unexpectedly, few studies have focused on other great apes despite evidence of proto-conversational rules in monkeys, thus preventing researchers from drawing conclusions on potential evolutionary origins of this behaviour. A previous study showed however that western lowland gorillas engage in soft call interactions that seem temporally- and socially-ruled. Indeed, interactions occurred mainly between individuals close in age who followed a preset response delay, thus preventing call overlap. Here, we experimentally investigated the presence of these rules in a captive gorilla group, using a violation-of-expectation paradigm. Head orientation responses suggest that the respect of response delay matters to subjects, but the importance of the interlocutors’ age proximity appeared less clear. The intensity of the response varied with subjects’ age in a context-dependent way, supporting a possible role of learning. Our findings support the growing number of studies highlighting the importance of vocal turn-taking in animals and a possible sociogenesis of this ability. The capacity to “converse” might have been a key step in the co-evolution of communication and complex sociality.

## Introduction

Despite the diversity of human cultures, some basic conversational rules are respected in all societies and thus appear universal^1^. These features are gathered in a so-called “contract of communication” that conversing interlocutors informally agree on^2^. The contract takes into account “context relevance” (*i.e*. the evaluation of the context as pertinent or not to initiate a conversation), “reciprocity” (*i.e*. the evaluation of both partners as valid interlocutors), and “contract-based temporal rules” (*i.e*. the respect of a reciprocal exchange of alternating, short, and flexible turns between two or more interlocutors^3^, and speech overlap avoidance). Several authors have questioned the possible biological bases of these conversational rules and have, for several decades, conducted cross-species comparisons to understand their origin and role across animal communication systems^4–7^.

To facilitate cross species investigations, authors typically distinguish “conversation-like vocal exchanges” from other vocal patterns like isolated calling (one call emitted and no other calls can be heard around, *e.g*. in red-capped mangabeys *Cercocebus torquatus*^8^), repeated calling (the same caller calls several times in a row, *e.g*. in Japanese macaques *Macaca fuscata*^9^), disorganized phonoresponses (one individual produces a call, typically an alarm call that triggers calls in an apparent chaotic way from the other group members, *e.g*. in blue monkeys *Cercopithecus mitis stuhlmanni*^10^), chorusing (two or more individuals overlap their emission of a given call type, *e.g*. in chimpanzees *Pan troglodytes*^11^) and duetting (two individuals synchronise long series of calls or songs with stereotyped temporal association: *e.g*. in birds^12–14^, and gibbons *Hylobates syndactylus*^15^). “Conversation-like vocal exchanges” are distinguished from the other types of vocal interactions because of their following specific features. First, they involve a diversity of recurrent vocal partners (which differs from duets). Second, interlocutors are typically familiar individuals belonging to a given social group and can be of any age or either sex (which differs from synchronized signalling^16–20^). Third, “conversation-like vocal exchanges” are not restricted to a specific context, which differs from – usually long-distance – collective communication associated with mate attraction, territory protection and environmental disturbance^21^, and time of the day or year (excluding morning choruses and reproductive signals^22,23^). Most commonly, these “conversation-like vocal exchanges” are observed in everyday and basically all day long short-distance communication (*i.e*. soft calls exchanged in peaceful interactions^5^), as in humans^3,24^.

The “conversation-like vocal exchanges” are temporally organized on the basis of turn-taking which refers to “the orderly exchange of purely communicative signals or behaviours between individuals characterized by principles for the coordination turn transfer, which result in observable temporal regularities” (cited from Pika and colleagues^25^). Communicative turn-taking has been found in a broad range of nonhuman species (*e.g*. elephants *Loxodonta africana*^26^; sperm-whales *Physeter macrocephalus*^27^; common bottlenose dolphins *Tursiops truncatus*^28^; starlings *Sturnus vulgaris*^4^; naked mole-rats *Heterocephalus glaber*^29^; bats *Diaemus youngi*^30^), including a broad range of nonhuman primates (NHP) species^25,31,32^. Thus, to respect turn-taking, interlocutors have to obey basic rules^5^. Interlocutors have coordinated roles, one of them sends a first signal and the other responds, and, when the conversation keeps going, the two interlocutors alternate. The respondent typically adjusts the timing of its vocal response by respecting a minimum silence gap (to prevent call overlap) and a maximum silence gap (to ensure call coordination). A recent study on marmosets, focusing on long periods of vocal interactions, showed such temporal coordination of vocal productions between individuals^32^.

Another important rule shared by human, nonhuman primates and several other animal species is that exchanging partners are not randomly nor opportunistically chosen (bottlenose dolphins^28^, large-billed crows *Corvus macrorhynchos*^33^, meerkats *Suricatta suricatta*^34^, sperm whales^27^, elephants^35^). Dunbar^36^ suggested that monkey call exchanges had started to function as a mean to groom-at-distance when the size of group became too large to allocate enough time to physical grooming among all members and maintain close bonds. Indeed, “conversation-like vocal exchanges” occur mainly between two interlocutors that are determined, according to the study species, by their affiliative bonds (humans^37^; NHP: spider monkeys *Ateles geoffroyi*^38^, Japanese macaques^39^, pygmy marmosets *Cebuella pygmaea*^6^, bonobos *Pan paniscus*^40^) or by their respective ages (humans^41^; NHP: common marmosets^42^, western lowland gorillas *Gorilla gorilla gorilla*^43^, Campbell’s monkeys^31^). In some cases, it was even found that human and nonhuman primates advertise this preferential bond by responding to one another using a matching acoustic structure, a phenomenon known as “vocal convergence” in ethology or “vocal accommodation” in sociolinguistics (humans^44^; NHP: spider monkeys^38^, Diana monkeys *Cercopithecus diana*^45^).

It is worth noting that some conversational characteristics have also been found in nonprimate species. However, only the vocal interactions of nonhuman primates match all the criteria defining a “conversation-like vocal exchange” (see above the mentioned features). The origins of such vocal behaviours in nonhuman primates remain debated, notably because most published research on the topic has focused on monkeys, and additionally available findings in nonhuman apes (phylogenetically closer to humans) are controversial. The current state of knowledge prevents to conclude between a convergent evolution guided by the requirements of social life or a shared inheritance, suggesting an ancient mechanism which was already present in the primate lineage.

To our knowledge, three studies have focused on vocal conversational rules in nonhuman great apes, two of which found support for turn-taking and call overlap avoidance (bonobos^40^; gorillas^43^), and one that did not (chimpanzees^46^). Authors also questioned the fact that these abilities to converse in animals are genetically programmed or, as in humans^47,48^, socially acquired. Empirical evidence is currently lacking to answer this question, although the few available studies on monkeys showed that juveniles break conversational rules more often than do adults (howler monkeys *Alouatta pigra*^21^; Campbell’s monkeys^49^). Moreover, playback experiments using a violation of expectation paradigm showed that adult monkeys clearly discriminated between conversationally appropriate and inappropriate vocal exchange patterns, whereas socially inexperienced juveniles did not (Japanese macaques^50^; Campbell’s monkeys^49^). These experiments show that the audience detects the violation of expected social rules in vocal interactions between third parties^49–52^, suggesting that the social rules in vocal interactions are not a simple neurobiological guided behaviour but a social awareness of the respect of social rules.

Here, we aimed to progress this debate by conducting a playback experiment with captive western lowland gorillas of different ages. A recent study, ran on a captive group of western lowland gorillas housed at the ZooParc de Beauval, found that individuals do seem to engage in socially- and temporally-ruled vocal interactions^43^. Vocal interactions typically involve grunts, defined as soft contact calls^53–55^ that are acoustically individually distinctive^55^. Grunts seem to play a key role in the coordination and peaceful activities, and thus act as a contact call^53–55^. During grunt exchanges, temporal rules are respected with an average “response” delay of 0.5 seconds (maximum 3 s) and with obvious call overlap avoidance^43^. Also, preferred interlocutors are non-randomly chosen. Grunt exchanges are mainly dyadic interactions that are most frequently observed among partners close in age, regardless of kinship^43^.

To assess the social relevance of these two potential conversational rules (inter-call durations and the age difference of interlocutors) for the audience in the captive population housed at the ZooParc de Beauval (n = 6), we compared the responses of gorillas to grunt exchanges that have been built to match both the temporal and the social rules (condition A), in comparison with built grunt exchanges which respectively violate the overlap avoidance expectation (condition B) and the expectation of an age proximity between vocally exchanging partners (condition C). We also tested whether the age of the subject (perceiver) influenced its responses to these different vocal exchanges. We expected a modification of the attentional state of the audience by comparing the difference of the total duration of head orientation and the total number of occurrences of locomotion in the direction of the loudspeaker in the 60 seconds after the onset of the second vocalisation and 60 seconds before playback. We also examined the first latency to reposition the head front, corresponding to the duration of the first gaze towards the loudspeaker after the diffusion. The change of attentional state may be in both ways: an increased response towards the non-congruent pattern (implying that animals are surprised to hear the violation of the overlap avoidance and the expectation of an age proximity between interlocutors, as in Japanese macaques^50^), or towards the natural ones that they are exposed to in their everyday social life (implying that the audience is strongly interested by a relevant interaction including their congeners, as in Campbell’s monkeys^49^).

## Results

**Figure 1:**
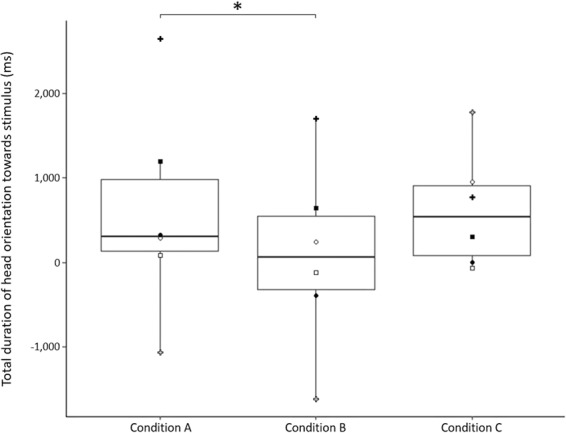
Total duration of head orientation toward the loudspeaker (in the 60 seconds ‘after’ minus 60 seconds ‘before’ playback) in the three playback conditions: “likely vocal exchange” (condition A), “overlapped vocal exchange” (condition B), “age-difference vocal exchange” (condition C). Each symbol corresponds to an individual. Box and whisker plots report the median, 25th and 75th percentiles, and the lowest and highest data, which are no more than 1.5 interquartile range from the box. *P < 0.05.

Analyses showed that individuals oriented their head towards the stimulus for a longer total time when temporal and social rules were respected (condition A) than when temporal rule was not (*i.e*. when calls were overlapping, condition B) (Wilcoxon signed rank test, V = 21, P = 0.031, Fig. 1). However, there was no significant difference in the duration of head orientations between the likely (condition A) and the age-difference (condition C) vocal exchange conditions (V = 12, P = 0.844), nor between the overlapped calls and the age-difference vocal exchange conditions (V = 8, P = 0.6875). Moreover, no effect of condition was found for latency to redirect the head after the first gaze in direction to the loudspeaker (condition A: mean ± SD = 2025 ± 1181msec; condition B: 2005 ± 72msec; condition C: 4995 ± 7256msec; V_A/B_ = 10, P = 1; V_A/C_ = 7, P = 0.563) nor for locomotion (condition A: 0.22 ± 0.29; condition B: 0.13 ± 0.31; condition C: 0.071 ± 0.21; V_A/B_ = 5, P = 0.423; V_A/C_ = 6, P = 0.174).

**Figure 2:**
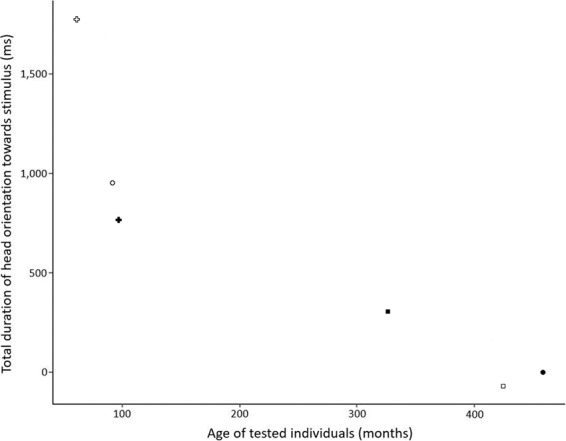
Total duration of head orientation in the “age-difference vocal exchange” (condition C) (in the 60 seconds ‘after’ minus 60 seconds ‘before’ playback) in relation to the subject’s age. Each symbol corresponds to an individual (same as in Fig. 1).

**Figure 3:**
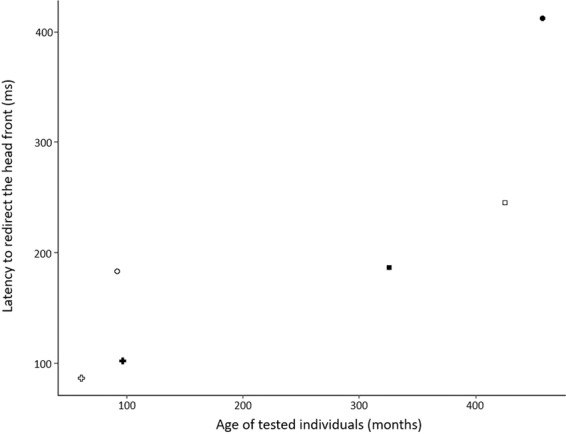
Latency to redirect the head front in the “likely vocal exchange” (condition A) in relation to the subject’s age. Each symbol corresponds to an individual (same as in Fig. 1).

The behavioural responses of the subject differed in some conditions according to its age. In the “age-difference vocal exchange” (condition C), we found that the older the subject, the shorter the head orientation duration towards the speaker (Spearman test, rs = −0.94, P = 0.017, Fig. 2). The total head orientation duration did not vary with subject’s age in the two other conditions (rs_A_ = 0.31, P = 0.564; rs_B_ = 0.03, P = 1). Likewise, when focusing on the latency to redirect the head after the first gaze directed to the speaker, we found that the older the subject, the longer the first head orientation in the “likely vocal exchange” (condition A) (rs = 0.94, P = 0.017, Fig. 3), but not in the two other conditions (rs_B_ = 0.49, rs_C_ = −0.49, P = 0.356 in both cases). However, the age of the subject did not predict locomotion (rs_A_ = −0.76, P = 0.08, rs_B_ = −0.13, P = 0.805, rs_C_ = 0.34, P = 0.512).

## Discussion

The violation-of-expectation paradigm is typically used in research on human and nonhuman animals to evaluate their ability to perceive certain rules^56^. Duration of post-playback head orientation responses is a common measure to assess a subject’s hearing general interest in a presented stimulus among both nonhuman^57,58^ and human^59^, notably newborn^60^ primates. In our study, results from the total duration of head orientation suggest that the gorilla’s audience pays attention to the vocal interactions among its group members and is more attentive to a “likely-vocal exchange” (condition A) than to an “overlapped vocal exchange” (condition B). In such “violation of attempt” setup, it is often predicted that unexpected stimuli would cause stronger responses than expected ones^61^. Indeed, nonhuman primates have shown more head orientations in response to a violation of the original pattern in several playback experiments^62,63^. Although our results may appear surprising in light of such studies, numerous authors have also reported an opposite effect of attempt violation on subjects’ responses. For instance, human infants tested in cross-modal studies show a familiarity/consistency preference^64,65^. The same is true for nonhuman primates. When exposing Japanese macaques to videos of conspecific facial expressions during vocal emissions, alongside the playback of a vocalisation which only matches one of the videos, subjects preferentially look at the congruent one (*i.e*. when vocalisation and video matched)^66^. A stronger attentional state towards an expected vocal sequence pattern rather than an unexpected one was also found in common marmosets^67^. Thus, the direction of the subjects’ attention bias in such experimental paradigms may be stimulus- or context-dependent. Interestingly, Lemasson and colleagues, who aimed answering the same research question as ours, obtained the same pattern in the Campbell monkeys they tested^49^.

In our study, the fact that the subjects paid a differential attention to the different test conditions (namely a strongest attention towards the “likely-vocal exchange” than the “overlapped vocal exchange”) suggests that the pattern was perceived and that temporal rules could be relevant to them. In the “overlapped vocal exchanges”, gorillas may not be able to extract all the relevant information, and notably not easily identify the callers, which can explain the weak responses of the audience in this condition. This can also be due to the fact that individuals evaluated the opportunity to participate in the ongoing simulated vocal exchange and lost interest quickly when they realised that the exchange was inappropriate, as suggested by Lemasson *et al*.^49^ after similar results obtained in adult Campbell’s monkeys. We cannot totally rule out other possible alternative interpretations (although not mutually exclusive), notably that the lack of attention towards the “overlapped vocal exchange” (condition B) could be due to the fact that this stimulus is shorter in duration (so with a smaller quantity of “acoustic” information). However, the short duration hypothesis is unlikely for two reasons. First, because the difference in mean duration of the stimuli respecting temporal rules (*i.e*. conditions A and C) and those not respecting them (*i.e*. condition B) is very small (see methods). Second, because we found no significant difference between conditions B and C, although their stimuli differ in duration. This also reinforces our idea that the possible message confusion associated with call overlapping is not the core origin of the attentional bias observed above. The avoidance of call overlap could be a relevant social rule in the interactions of western gorillas, but did not elicit an attentional response strong enough to induce locomotion toward the loudspeaker. This is also true for the other conditions and not surprising given the fact that the simulated vocal exchanges involved soft contact calls emitted during peaceful activities^53–55^. Anyhow, such importance of the temporal organization of vocal interactions has already been highlighted in several monkey species^25,49^. In Campbell’s monkeys and Japanese macaques respectively, individuals that violate the turn-taking rule are typically young and inexperienced juveniles^49^ or low-ranking (socially isolated) adult males^68^. In most human cultures, conversational overlap denotes a conversation failure and can lead to the end of an exchange^3^. In fact, vocal overlapping is perceived as a serious impoliteness in many traditional human societies^69^. In birds as well, overlap increases with aggressiveness and may lead to the end of an exchange (for a review see^4^). For example, in black capped chickadees (*Poecile atricapillus*), dominant males overlap more than do subordinates^70,71^ and this overlapping increases arousal in overlapped individuals^72,73^.

The importance of age proximity between interlocutors for a gorilla audience remains unclear. Indeed, the age proximity or the age distance between interlocutors did not elicit different responses at the group level. One possible explanation is that breaking the social (age difference) rule is twice more common (15%) than breaking the temporal (overlap) rule (8%) in gorillas and thus less surprising. The systematic focus on the silverback adult male in all broadcast vocal exchanges could have however induced a bias of general interest (*i.e*. silverback male vocalizations would systematically attract the attention of his groupmates whichever the context they are produced in), given that the role of the adult male is central in the social cohesion and coordination of the group^74,75^. This dominant male indeed develops tight bonds with each individual and is a key interlocutor for all group members. This supposed social rule (based on observations of naturally produced vocal exchanges^43^) cannot be considered as mandatory at this stage given that it does not elicit an unanimous behavioural response to violation.

Interestingly, the behavioural responses of the audience varied between individuals according to their age. The total duration of head orientation shows that the stronger attentional state of young gorillas toward the age-difference vocal interaction (*i.e*. condition C) is not trivial, and could reveal their interest for an eventual interaction involving another young individual and the adult male. That could explain why we did not find similar correlations in “likely vocal exchange” and “overlapped vocal exchange” conditions (*i.e*. conditions A and B) involving only adult individuals. This result suggests that, even if not a mandatory rule, gorillas may have expectations in this regard. Moreover, when we considered the first latency to reposition the head front as an indicator of the level of interest towards the stimulation, older gorillas paid more attention to exchanges between interlocutors close in age respecting common response delay (condition A) than did younger gorillas. Older gorillas may be socially more experienced than younger ones and more reliably perceive if a vocal interaction is relevant, as the “likely vocal exchange (*i.e*. condition A), or not, as the “overlapped vocal exchange” and the “age-difference vocal exchange” (*i.e*. conditions B and C), explaining the absence of correlation in conditions B and C. Nonetheless, these findings support the prediction, earlier suggested in monkeys^49^, that appropriate rules of conversing may be socially learned. Differences between adult and young individuals regarding the appropriateness of the context of call emission have already been reported in various primate species (see food calling in tamarins *Saguinus oedipus*^76^, and alarm calling in vervet monkeys *Chlorocebus pygerythrus*^77^). Some studies have also showed vocal development in the ability to interact properly with congeners (see vocal synchronisation in gibbons *Hylobates agilis agilis*^78^, as well as call matching and choice of preferred interlocutors in spider-monkeys^38^). Moreover, two playback studies have confirmed that conversational rules can be detected by adults and not by juveniles (turn-taking in Campbell’s monkeys^49^, and call matching in Japanese macaques^50^).

We acknowledge that our study is just one step towards a more advanced understanding of vocal interactions in great apes, and given the small sample size, some factors other than age might play a role. Indeed, despite all the advantages that the captivity offers to the study of vocal communication, gorillas cannot be isolated during experiments because of the risk of intense stress. Despite the randomisation of subject and condition orders and despite the fact that playbacks were time-spaced (over two months), a habituation effect cannot be discarded. Lastly, our result showing that young individuals responded strongly towards the “age-difference situation” (*i.e*. condition C) may suggest that the attentional response between each condition was driven by the age of individuals. Then, this difference of attentional state of the youngers towards simulated vocal exchanges involving individuals of their own age range could induce a bias, mainly in the “overlapped vocal exchange” condition which include only adult individuals. To address this possible bias, a supplementary condition may be considered in future comparable studies. It would be similar to the “overlapped vocal exchange” but involving a young individual as first emitter who vocally interacts with the adult male, as the simulated vocal exchanges played back in this study (condition C). Our results are nevertheless promising and seem pointing out the possible existence of conversational rules managing vocal interactions in western lowland gorillas^43^. Future studies may consider investigating multiple and larger groups with a greater age-category range, and may test for potential intergroup variations. In the same vein, Levréro and colleagues^40^ highlighted that bonobos are able to spontaneously display primitive conversation rules guided by social bonds, and Lameira and colleagues^79,80^ showed that orang-utans (*Pongo pygmaeus*) have the cognitive abilities to produce spontaneous vocal exchanges. Only one study in apes, conducted in the field on male chimpanzees, revealed that the production of long calls (*i.e*. pant-hoots) is non-temporally organized (*i.e*. emissions are rather isolated calls without consecutive calls from other group members, or cases of synchronous choruses^46^). A recent study however has showed that chimpanzees have the ability to engage in gestural turn-taking^81^. All these findings suggest that all great apes possess the cognitive skills to engage in a “conversation-like” interaction. These results are promising and deserve further comparative work.

In conclusion, these recent studies on great apes highlight how vocal interactions are governed by social rules in our closest relatives, as they are across human cultures. Our findings help to fill the gap between nonhuman and human primate communication, particularly in terms of our understanding of factors that shape vocal interactions. The increase of the complexity of social interactions (from birds to mammals including monkeys, nonhuman apes and humans) may be at the origin of the most complex rules governing human communication. Parallels between human language and nonhuman primate vocal communication appear multifaceted, *e.g*., information coding in acoustic structure, syntactic structure and socially-determined plasticity^49,82,83^. Here, a vast research area is opening into the developmental acquisition of conversational rules. The function, as well as the content of information in vocal exchanges remains unclear and still needs to be explored more systematically. Yet what is clear, is that the capacity to interact vocally with others and to respect ruled vocal interactions could have been an important step in the evolution of the vocal communication of primates^36^. This ability appeared before the capacity to articulate sounds in speech and to learn new acoustic structures, and is thus likely rooted deep in the animal lineage.

## Methods

### Compliance with ethical standards

All applicable international, national, and/or institutional guidelines for the care and use of animals were followed. The study has been conducted in accordance with the current laws in France (agreement with the 2010/63/UE). Animal husbandry and care were under the management of the animal caregivers of the ZooParc de Beauval, France. Our study was based on non-invasive observations and was approved by the ethics committee “Comité Rennais d’Ethique en matière d’Expérimentation Animale” (*i.e*. Rennes Ethical comity for experiments using animals; CNREEA 2018041710224608-APAFIS#14920).

### Animals and housing conditions

The sample comprised twelve captive-born western lowland gorillas, including one adult silverback male, four adult females, and seven (four males and three females) immature individuals. The composition of the captive group reflects that of wild groups^84,85^. All adult females were unrelated. The adult male had sired all the immature individuals of the group.

**Figure 4:**
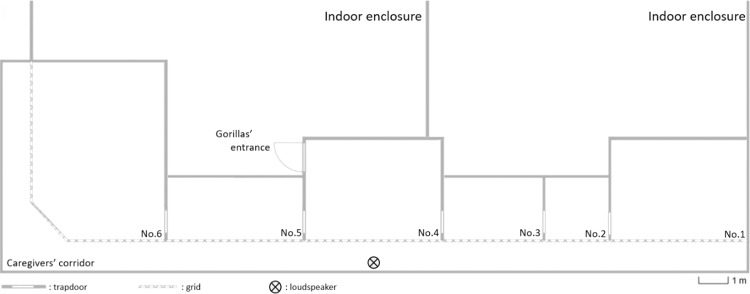
Diagram of the layout of the cages.

The animals were housed at the ZooParc de Beauval (France) in an enriched building of 720 m^2^ (6 m high, with glass walls, wired roof, straw litter and wood perches, as well as temperature and humidity control), connected to several adjacent cages totalling 59 m^2^ (average size: 5 m^2^) with visual and auditory access between them. Cages were aligned from right to left (*i.e*. from No. 1 to No. 6), and cage No. 4, of the silverback male, was central (Fig. 4). Depending on the weather conditions, group had access to a 3,500 m^2^ outdoor enclosure (with grass, bush, and wood perches). Gorillas shared the enclosure with eight patas monkeys (*Erythrocebus patas*).

Gorillas were trained to enter the cages daily for breakfast and medical-training between 8:30 and 10:30 a.m., returning afterward to the large enclosure in which they spent the rest of the day and night. While they were free to go in any cages, each gorilla used to settle every day in a determined cage (one individual per cage, except for mother and offspring who may stay together). Once they were in their usual preferred cage, caregivers closed the trapdoors between each cage in order to avoid food theft (see Table 1 for cage occupation).

**Table 1:** Tested individuals in the payback experiment.

Subject name (birth date)	Age-sex category^a^	Mother’s name (all fathered by the adult male)	Cage number (date and new cage number if changed during the experimental period)	Number of calls used in built vocal exchanges
Inge (02/03/1980)	Adult female		No. 1	4
Kabinda (10/12/1982)	Adult female		No. 5	4
Sheila (18/02/1991)	Adult female		No. 6	4
Mapenzi (14/04/2010)	Subadult male	Kabinda	No. 3	0
Kuimba (19/08/2010)	Subadult female	Tamarilla^†^	No. 2	2
Mayélé (21/03/2013)	Juvenile female	Kabinda	No. 5 (05/11/2018, No. 3)	2

^a^Age-sex categories defined according to Gatti and colleagues^89^; ^b^Loudspeaker location; ^†^Dead individual (10/02/2017).

### Vocal recordings

Between April and July 2018, we recorded 474 spontaneous grunts, produced by the twelve individuals during 33 breakfast sessions for a total of 27 hours of recording, using all occurrence sampling^86^. We pooled together all possible grunt structures (*i.e*. atonal and tonal grunts, single and double grunts^53,54^). Recordings were done at an average distance of 2 meters using a Marantz PMD660 handheld digital audio recorder (Marantz, Japan) (sample rate 44.1 kHz, resolution 32bits, wav. format) connected to a directional Sennheiser MKH70-1 microphone (Sennheiser, Germany). We identified 125 vocal interactions, based on Lemasson and colleagues’ definition^43^ for gorillas (*i.e*. series of calls emitted by different callers with an inter-call duration of less than 3 seconds). Vocal interactions typically involved callers from different cages (usually only two callers: 77%) and non-overlapped exchanged calls (92%). The adult male contributed 70% of these vocal interactions and was significantly more often a respondent than an initiator (Binomial test, N_1rst position_ = 20, N_2nd position_ = 41, p = 0.01). He responded more often to adult females (85% of response) than to young individuals (*i.e*. sub-adults, juvenile and infants, 15%).

### Playback experiments

Playback experiments took place between September and December 2018. Participating gorillas never experienced any playback experiment in the past. Here, we tested their responses to the broadcast of three types of vocal exchanges, *i.e*. one control condition, one violating the temporal expectation and one violating the social expectation:

- Condition A, “likely vocal exchange”: we built a control dyadic vocal exchange (as described in Lemasson *et al*.^41^) between two individuals close in age (*i.e*. both adults), that respected the average inter-call duration of 500 milliseconds (ms) (see Supplementary Fig. S1);
- Condition B, “overlapped vocal exchange”: we built an unlikely vocal exchange that did not obey the temporal turn-taking rule, with 50% of overlap between two vocalisations^87^ emitted by two adults (see Supplementary Fig. S2);
- Condition C, “age-difference vocal exchange”: we built an unlikely vocal exchange, with an inter-call duration of 500 ms, that did not obey the social rule with two callers of distant ages (*i.e*. initiator: sub-adult or juvenile, mean age ± SD = 6.39 ± 1.33 years; respondent: adult male, 27 years; see Supplementary Fig. S3).

Vocal exchange acoustic stimuli were created using the vocalisations recorded during the aforementioned observations, discarding non-exchanged grunts, and using PRAAT and Goldwave softwares. Calls of initiators and respondents were systematically concatenated to respect their original position from dyadic natural vocal interactions previously recorded. Even the control dyadic “likely vocal exchanges” were built to prevent any bias due to artificial concatenation of calls. To match the most frequent vocal events occurring in natural conditions, we decided to use the adult male as the respondent in all acoustic stimuli and different females as initiators. To prevent kin-bias, subjects only heard unrelated female initiators. Thus, a given adult female gorilla was allotted to a given subject in conditions A and B. To prevent pseudo-replication, a given call exemplar was used only once. Given that spontaneous vocal interactions (*i.e*. abovementioned recordings) contain an equal number of grunts and double grunts, we used 50% of grunts and 50% of double grunts. The total durations of the played back stimuli in conditions A and C (excluding inter-call durations) were respectively 163 ± 26 ms and 146 ± 30 ms, and 125 ± 36 ms in condition B.

Experiments took place during the usual breakfast in the corridor facing the cages. Sounds were played using a TAG Premio8.2 loudspeaker connected to the Marantz player at 60 dB SPL. The intensity of 60 dB corresponds to the grunts recorded in this group in similar conditions at an average distance of 2 meters. This intensity threshold matches the intensity used in other nonhuman primate studies (*e.g*. Japanese macaques^50^,bonobos^88^).The loudspeaker was systematically placed before the arrival of gorillas in the cages at 1 meter in front of cage No.4 (of the silverback male, see Fig. 4) and could not be seen by the other gorillas. The experimenter (LP) sat in front of the tested animal and fed it routinely with fruits, dry-food and lastly vegetables (always in this order), through the wire mesh. The camera was positioned behind the experimenter, above his head. The breakfast typically lasted around 30 minutes and the playback occurred at mid-term. The experimenter offered food to the subject in order to control the central position of his body and head at T0 (*i.e*. playback time). Before broadcasting the stimuli, some conditions had been respected to make the situation as plausible as possible: (*i*) both initiators and respondents used in a stimulated vocal exchange on the same side of the loudspeaker, both at the opposite side of the tested individual, and were not visible from the subject point of view, (*ii*) no vocalisation was heard within the two minutes preceding the playback. To prevent any possible influence from the experimenter, the experimenter’s gaze had to focus on the gorilla’s chest during the entire feeding period. To habituate animals to the experimental setting, we ran ‘silent sessions’ during which the full equipment was set up to mimic the conditions of real playback sessions but without any sound broadcast. Twenty days before starting the first playback session, we performed ‘silent sessions’ once a day, every day. We then performed randomly ‘silent sessions’ throughout the entire duration of the experiment. To limit habituation, no more than one subject was tested per day and each subject was tested at average intervals of 27 days (min: 3, max: 73 days apart). This variation is due to the fact that we had to stop our experiments on two occasions, after 11 and 28 days respectively, because of on-going conflicts in the group or construction work in the enclosure.

Six individuals were tested in the playback paradigm (Table 1). The three infants were excluded from the study as they were still dependent on their mothers. The silverback male was not tested as he was involved in the creation of all acoustic stimuli. We discarded one adult female and one subadult female, because they were too stressed during the experiments, either by the presence of the experimenter or by a conflict with other group members. Each tested gorilla heard each of three distinct conditions, resulting in 18 (3 ×6) stimuli in total. Subject order and condition order were both randomized.

### Recording of behavioural responses

We filmed the tested gorilla’s behaviours before and after playback. All videos were analysed in slow motion (scale of millisecond) using Kinovea 80.8.15 software for coding, while the experimenter was ‘blind’ to the experimental condition. The behavioural responses measured were the difference in the total duration of head orientation and in the total number of occurrences of locomotion (already observed in bonobos towards similar stimuli^87^) in the direction of the loudspeaker before and after the playback. The time windows used for the measurements were 60 seconds before and 60 seconds after the onset of the second vocalisation (*i.e*. T0 = onset of male calling, as this is the moment when subjects typically detect the breaking of the rule, as in Bouchet and colleagues^50^). This correction was chosen because contact call exchanges are not rare events that would typically trigger a strong surprise, because inter-cage gaze exchanges are frequent at breakfast time and lastly because subjects differed in their baseline interest towards their social environment; we thus wanted to capture not only the degree of interest towards the playback area but more importantly the increase/decrease of interest towards this area, as in other previous studies^57,90,91^.We also measured the first latency to reposition the head front (*i.e*. facing front the experimenter), corresponding to the duration of the first gaze towards the loudspeaker (starting the measure at T0 if the individual turned its head before the onset of the second vocalisation).

Normality and homogeneity of variance were assessed by inspecting residuals following Shapiro-Wilk W tests. Given the non-normally distributed data, we ran non-parametric tests^92^ with a significance threshold of 0.05. The difference of total duration of head orientation, of locomotion occurrences, and the latencies to reposition the head between likely (condition A) and unlikely vocal exchanges (conditions B and C) were tested using exact Wilcoxon signed-rank tests. Spearman’s correlation tests were then run to investigate the possible influence of the age of the subjects on their responses to each situation. Data were analysed in R Studio0.99.903 (R version3.3.1: R Core Team2016).

## Supplementary information

Supplementary information.

## Data Availability

The data generated and analysed during the current study are available from the corresponding author on reasonable request.
