# Supplementary material for: Breaking conversational rules matters to captive gorillas: A playback experiment
Source: Sci Rep. 2020 Apr 24;10:6947. doi: 10.1038/s41598-020-63923-7 (PMC7181860; doi:10.1038/s41598-020-63923-7)
Supplement: Supplementary file 1 — Supplementary information. [file 41598_2020_63923_MOESM1_ESM.pdf]

Breaking conversational rules matters to captive gorillas: a playback experiment

Loïc Pougnault[1,2,3], Florence Levréro[2]*, Baptiste Mulot[3], Alban Lemasson[1]*

[1] Univ Rennes, Normandie Univ, CNRS, EthoS (Éthologie animale et humaine) - UMR 6552, F-35000 Rennes, France.

[2] Université de Lyon/Saint-Etienne, CNRS, Equipe Neuro-Ethologie Sensorielle, ENES / CRNL, UMR5292, INSERM UMR_S 1028, Saint-Etienne, France.

[3] ZooParc de Beauval & Beauval Nature, 41110 Saint Aignan, France

* Same contribution

Supplementary materials

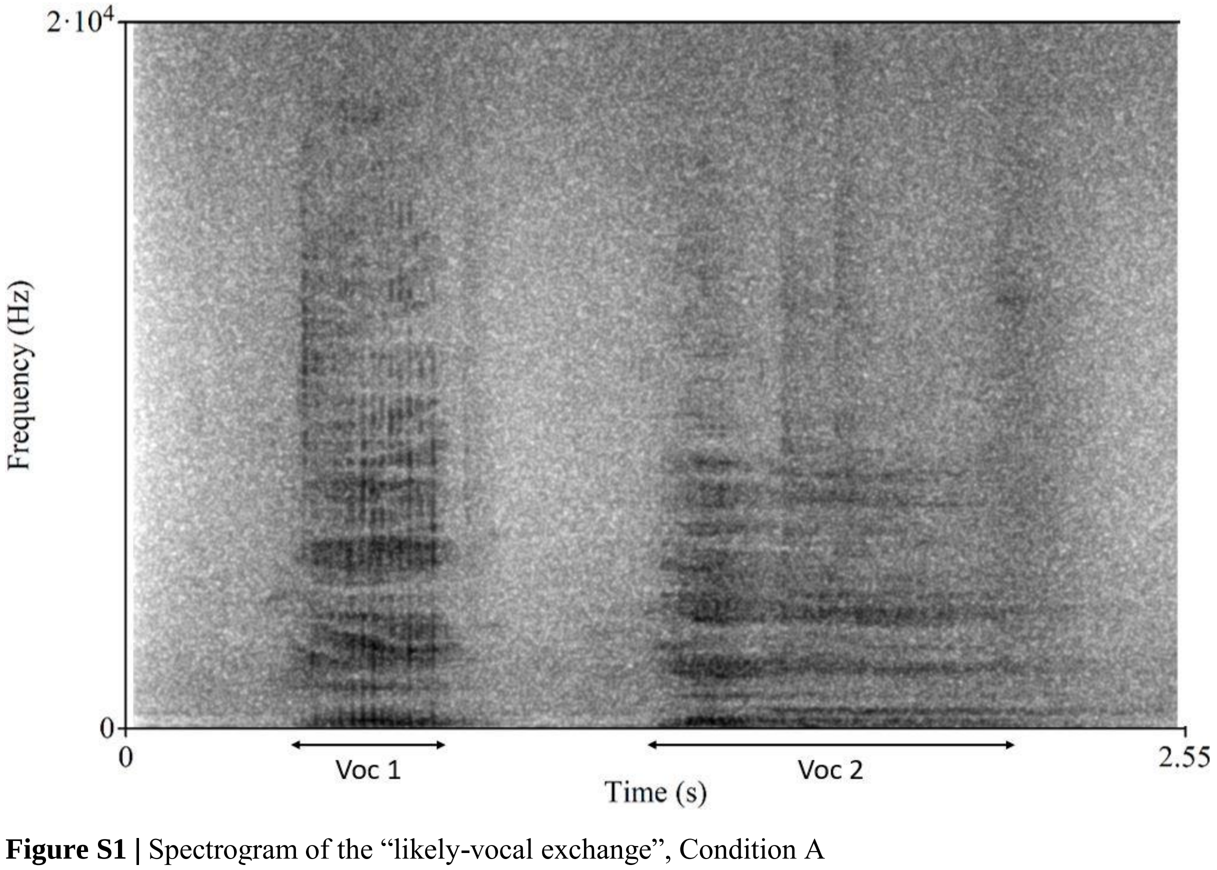

Figure S1 | Spectrogram of the “likely-vocal exchange”, Condition A

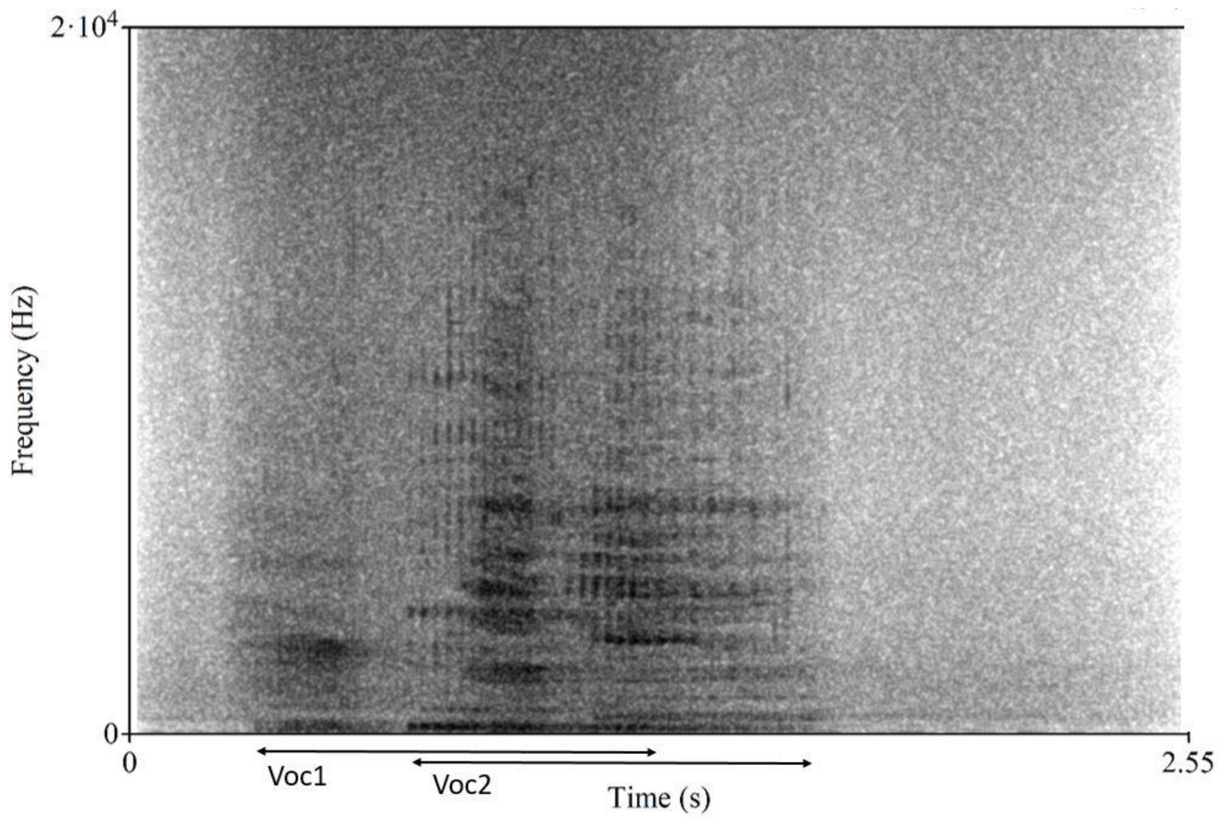

Figure S2 | Spectrogram of the “overlapped vocal exchange”, Condition B

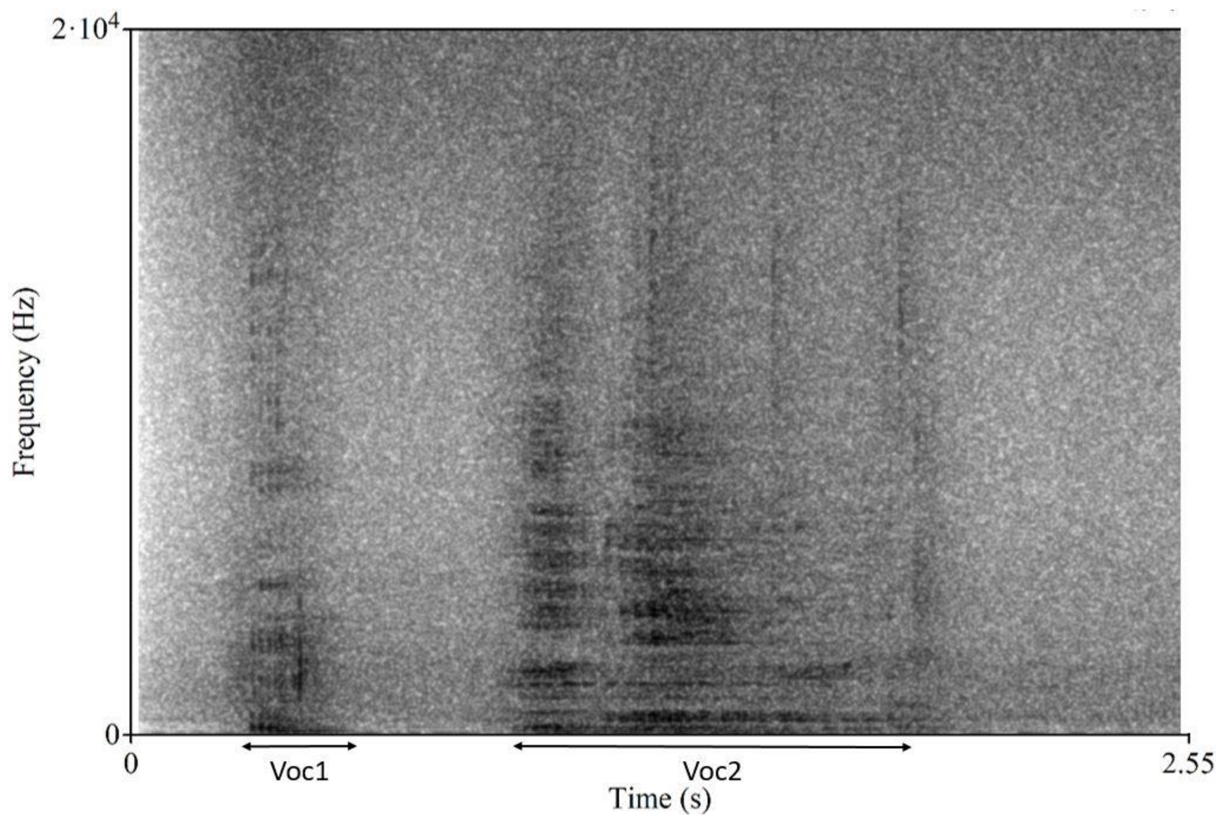

Figure S3 | Spectrogram of the “age-difference vocal exchange”, Condition C
